# Pollution indices as useful tools for the comprehensive evaluation of the degree of soil contamination–A review

**DOI:** 10.1007/s10653-018-0106-z

**Published:** 2018-04-05

**Authors:** Joanna Beata Kowalska, Ryszard Mazurek, Michał Gąsiorek, Tomasz Zaleski

**Affiliations:** Department of Soil Science and Soil Protection, Institute of Soil Science and Agrophysics, University of Agriculture, Al. Mickiewicza 21, 31-120 Kraków, Poland

**Keywords:** Heavy metals, Pollution indices, Geochemical background, Different soil uses

## Abstract

The paper provides a complex, critical assessment of heavy metal soil pollution using different indices. Pollution indices are widely considered a useful tool for the comprehensive evaluation of the degree of contamination. Moreover, they can have a great importance in the assessment of soil quality and the prediction of future ecosystem sustainability, especially in the case of farmlands. Eighteen indices previously described by several authors (*I*_geo_, PI, EF*, C*_*f*_, PI_sum_, PI_Nemerow_, PLI, PI_ave_, PI_Vector_, PIN, MEC, CSI, MERMQ, *C*_deg_, RI, mCd and ExF) as well as the newly published Biogeochemical Index (BGI) were compared. The content, as determined by other authors, of the most widely investigated heavy metals (Cd, Pb and Zn) in farmland, forest and urban soils was used as a database for the calculation of all of the presented indices, and this shows, based on statistical methods, the similarities and differences between them. The indices were initially divided into two groups: individual and complex. In order to achieve a more precise classification, our study attempted to further split indices based on their purpose and method of calculation. The strengths and weaknesses of each index were assessed; in addition, a comprehensive method for pollution index choice is presented, in order to best interpret pollution in different soils (farmland, forest and urban). This critical review also contains an evaluation of various geochemical backgrounds (GBs) used in heavy metal soil pollution assessments. The authors propose a comprehensive method in order to assess soil quality, based on the application of local and reference GB.

**Electronic supplementary material:**

The online version of this article (10.1007/s10653-018-0106-z) contains supplementary material, which is available to authorized users.

## Introduction

The problem of high heavy metal content in soils is related to the latter’s geo- and bioaccumulation ability (Fagbote and Olanipekun 2010; Gong et al. 2008; Hong-gui et al. 2012; Ogunkunle and Fatoba 2013; Oti Wilberforce 2015; Pejman et al. 2015; Sadhu et al. 2012) as well as the transport rate within the soil profile (Fagbote and Olanipekun 2010). Distribution of heavy metals within the soil profile could provide information about their origin (Chen et al. 2015; Pejman et al. 2015; Sołek-Podwika et al. 2016). Soil enrichment with heavy metals could reflect historical human activities (Shu and Zhai 2014; Sołek-Podwika et al. 2016; Tang et al. 2015; Mazurek et al. 2017). On the other hand, the present anthropogenic pollution sources, such as transport, industry and agriculture, have an undoubted influence on heavy metal accumulation in the soil (Gao and Chen 2012; Ogunkunle and Fatoba 2013; Sayadi et al. 2015). Heavy metals can be derived from both local and distant sources of emissions, and therefore can be deposited in situ or, due to their ability to be bound by dust, can be transported over long distances (Mohamed et al. 2014; Ripin et al. 2014; Mazurek et al. 2017). Most anthropogenic pollutants are emitted into the atmosphere and then are deposited on the soil surface (Liu et al. 2016; Ripin et al. 2014). Accumulation of metals may also be supported by natural processes. Heavy metals are considered substantial constituents of the Earth’s crust (Grzebisz et al. 2002; Hawkes and Webb 1962; Rudnick and Gao 2003; Zhou et al. 2015); hence, the nature of the parent material and pedogenesis at the site can create favorable or unfavorable conditions for heavy metal accumulation. Furthermore, weathering of the parent material is a natural process affecting the amount of heavy metals in the soil (Chen et al. 2015; Kierczak et al. 2016).

The problem of high concentrations of heavy metals, especially in agricultural soils, creates a global environmental issue due to the crucial importance of food production and security (Chen et al. 2015; Kabata-Pendias 2011; Kelepertzis 2014). Incorporation of heavy metals into the trophic chain may affect animal and human health (Al-Anbari et al. 2015; Begum et al. 2014; Gao and Chen 2012; Mohamed et al. 2014; Mmolawa et al. 2011; Pejman et al. 2015; Sadhu et al. 2012; Varol 2011; Zhang et al. 2012). Growing awareness of ever-expanding industrialization as well as intensive agricultural soil use and their influence on the content of heavy metals in the soil necessitates the appropriate evaluation as well as determination of their ecological risk (Baran et al. 2018, Gao and Chen 2012; Håkanson 1980; Kowalska et al. 2016; Zhong et al. 2010). Heavy metal pollution is visible in urban centers and farmland located in the vicinity of pollution sources, but also occurs outside these areas as well (Al-Anbari et al. 2015). Analysis of studies of time trends of heavy metal content in soils allows the tracing back of the development of industrialization as well as the use of fertilizers in the last decades. This clearly shows that there is a permanent tendency toward increased heavy metal accumulation (Al-Anbari et al. 2015; Gong et al. 2008; Hu et al. 2013; Wang et al. 2015). Therefore, it is necessary to use accurate and precise instruments in order to detect and, as far as possible, stop progressive soil degradation (Gong et al. 2008).

Numerous geochemical studies have contributed to the creation of an extensive database of heavy metal background values that can now be used for the evaluation of environmental quality (Gong et al. 2008; Obiora et al. 2016; Rodríguez et al. 2013; Wei and Yang 2010; Wu et al. 2015; Xia et al. 2011). However, analysis of the total contents of heavy metals in the soil may not always be a sufficient method of assessment (Caeiro et al. 2005; Hong-gui et al. 2012; Kowalska et al. 2016; Long et al. 1995). Therefore, for the assessment of heavy metal enrichment and its relationship with soil properties many computational tools have been applied (Gong et al. 2008; Mazurek et al. 2017). The total content, as well as statistical mechanisms and the relationship between the content of heavy metals and soil properties, such as correlation or regression, does not provide comprehensive information on the degree of soil contamination (Kowalska et al. 2016; Liu et al. 2016). In the case of comparisons of the content of heavy metals to the limiting values given in the literature, it is possible to only approximately determine the probability of contamination and this does not provide holistic information on the state of soil quality (Caeiro et al. 2005; Jiang et al. 2014; Nannoni and Protano 2016; Zhiyuan et al. 2011).

The key to the effective assessment of soil contamination with heavy metals lies in the use of pollution indices (Table 1). One of the first indices was created by Müller (1969) and Håkanson (1980). Pollution indices can be regarded as a tool and guide for a comprehensive geochemical assessment of the state of the soil environment (Caeiro et al. 2005; Dung et al. 2013; Gong et al. 2008; Kowalska et al. 2016; Mazurek et al. 2017). The comprehensive nature of assessing soil quality through the use of indices is also demonstrated by the opportunity it affords to estimate environmental risk as well as the degree of soil degradation (Adamu and Nganje 2010; Caeiro et al. 2005). The indices help to determine whether the accumulation of heavy metals was due to natural processes or was the result of anthropogenic activities (Caeiro et al. 2015; Elias and Gbadegesin 2011; Gong et al. 2008; Sutherland 2000). Further, pollution indices have a great importance for monitoring soil quality and ensuring future sustainability, especially in the case of agro-ecosystems (Ogunkunle and Fatoba 2013; Kelepertzis 2014; Ripin et al. 2014).

**Table 1:** Indices of heavy metal pollution given in the literature, their scope, strengths and weaknesses

Index	Application scope	Strengths	Weaknesses	Author(s)
Individual				
*I*_geo_	Assessment of the pollution levels in soil of individual heavy metals	Allows the comparison of the present and previous contamination Widely used Simple quantity method GB application 1.5 multiplication factor reduces possible variation of lithogenic effects Precise scale	Incorrect choice of GB leads to mistaken results Possible natural fluctuation within the GB Omits the available ability of heavy metals Does not take into account natural geochemical variability	Abrahim and Parker (2008), Begum et al. (2014), Chen et al. (2015), Dung et al. (2013), Fagbote and Olanipekun (2010), Gong et al. (2008), Guan et al. (2014), Ghazaryan et al. (2015), Grzebisz et al. (2002), Inengite et al. (2015), Karim et al. (2015), Kouamé et al. (2013), Li and Yang (2008), Liu et al. (2016), Loska et al. (2004), Mmolawa et al. (2011), Mohamed et al. (2014), Nikolaidis et al. (2010), Ololade (2014), Omatoso and Ojo (2015), Sayadi et al. (2015), Sadhu et al. (2012), Su et al. (2014), Tang et al. (2015), Wang et al. (2015), Varol (2011) and Zhiyuan et al. (2011)
PI	Evaluation of the degree of individual heavy metal contamination in topsoil	Easy to apply (calculated based the ratio between concentration in topsoil and GB values) Widely used GB application Precise scale	Does not require the variation of natural processes Omits the available ability of heavy metals The key is choice of appropriate GB	Al-Anbari et al. (2015), Begum et al. (2014), Chen et al. (2015), Gong et al. (2008), Hong-giu et al. (2012), Hu et al. (2013), Jiang et al. (2014), Karim et al. (2015), Li and Yang (2008), Mohamed et al. (2014), Mmolawa et al. (2011), Ogunkunle and Fatoba (2013), Ololade (2014), Ripin et al. (2014), Sadhu et al. (2012), Sayadi et al. (2015), Zhong et al. (2010) and Varol (2011)
EF	Effective tool for heavy metal content comparison Determination of heavy metal origin	Estimates anthropogenic impact Assessment of heavy metal origin Reduces metal variability Standardization of the element measures against one that is used for low occurrence variability elements Assessment of the pollution by individual heavy metals Precise scale	Measured with respect to reference values Evaluate above uncontaminated concentration The key is choice of appropriate GB	Abrahim and Parker (2008), Fagbote and Olanipekun (2010), Gong et al. (2008), Hu et al. (2013), Inengite et al. (2015), Karim et al. (2015), Mohamed et al. (2014), Mmolawa et al. (2011), Nikolaidis et al. (2010), Ololade (2014), Omatoso and Ojo (2015), Sadhu et al. (2012), Sayadi et al. (2015), Sutherland (2000), Wang et al. (2015), Varol (2011) and Zhong et al. (2012)
*C*_f_	Evaluation of soil quality Help to describe toxic substances	Simple and direct method Individual for each metal Comprising the difference between sample and reference values Obtained by dividing the concentration of each metal Precise scale	Does not require the variation of natural processes Omits the available ability of heavy metals Does not include GB Pre-industrial reference value is necessary	Håkanson (1980), Inengite et al. (2015) and Loska et al. (2004)
BGI	Evaluates the biosorption degree of contaminants	Shows vertical mobility of heavy metals Easy to calculate Precise scale	Does not require the variation of natural processes Omits the available ability of heavy metals	Mazurek et al. (2017)
Complex				
PI_sum_	Assesses the overall contamination of heavy metals group	Combines all analyzed heavy metals Allows comparison of the pollution in different soil ecosystems Based on *PI* values	Does not require the variation of natural processes Omits the available ability of heavy metals The key is choice of appropriate GB Lack of precise scale	Håkanson (1980), Inengite et al. (2015) and Loska et al. (2004)
PI_Nemerow_	Assessment of the overall quality of soil	Directly reflects the soil environment pollution Highlights the most contaminated elements GB values, threshold as well as baseline values may be used Widely used Takes into account all individual elements Precise scale Based on *PI* values	Does not include weighing factor Needs to rank elements	Al-Anbari et al. (2015), Gong et al. (2008), Guan et al. (2014), Hong-giu et al. (2012), Hu et al. (2013), Inengite et al. (2015), Ogunkunle and Fatoba (2013), Oti Wilberforce (2015), Shu and Zhai (2014) and Zhong et al. (2010)
PLI	Assessment of the level of contamination/extent of heavy metal	Combines any number of analyzed heavy metals Easy to apply Widely used Allows comparison of the pollution in different soil sites GB application Represents the number of times by which heavy metal exceeds its natural content in soil Based on *PI* values	With respect to GB Does not require the variation of natural processes Omits the availability of heavy metals	Begum et al. (2014), Karim et al. (2015), Kouame et al. (2013), Mohamed et al. (2014), Mmolawa et al. (2011), Ololade (2014), Pejman et al. (2015), Sadhu et al. (2012), Sayadi et al. (2015), Varol (2011)
PI_avg_	Evaluation of soil quality due to contamination	Easy to apply Lack of threshold for maximum values Based on *PI* values	Measured with respect to reference values Not widely used Takes into account the average No precise scale	Gong et al. (2008) and Inengite et al. (2015)
PI_Vector_	Overall assessment of heavy metal accumulation	Easy to calculate GB application Based on *PI* values Combines any number of contaminations in one index	Not much use in the literature Depends on *PI* values Does not require the variation of natural processes No precise scale	Gong et al. (2008)
PIN	Overall assessment of heavy metal	Easy to calculate All contamination is integrated into a single value Precise scale Based on *PI* values	Not widely used Does not consider natural geochemical processes Computation of *PI*_*Class*_ is necessary	Caeiro et al. (2005) and Gong et al. (2008)
MEC	Allows comprehensive assessment, including series of heavy metals	Easy to apply Gives information about heavy metal origin Takes into consideration all studied heavy metals	Kloke (1979) values are required Not widely used Does not consider natural geochemical processes No precise scale	Adamu and Nganje (2010) and Pejman et al. (2015)
CSI	Assessment of the intensity of heavy metal accumulation	Helpful to determine the limit of toxicity All contamination is integrated into a single value Includes adverse biological effects Precise scale	Not widely used Requires values for ERM and ERL Needs weight of every heavy metal	Pejman et al. (2015)
MERMQ	Tool for recognizing harmful effects of heavy metals Assessment of pollution risk level	Application for reducing a large amount of pollutants into one index Can prioritize regions of potential hazards Helps assess biological effects Precise scale	Not much used in the literature ERM values are required Does not require the variation of natural processes	Gao and Chen (2012) and Pejman et al. (2015)
*C*_deg_	Evaluates the degree of contamination in soil	The number of analyzed heavy metals is not limited Assesses a sum of contamination factors Precise scale	Not widely used Does not consider natural geochemical processes Does not include GB Pre-industrial reference value is necessary	Håkanson (1980), Inengite et al. (2015) and Loska et al. (2004)
RI	Evaluation potential ecological risk from heavy metals Contamination assessment	Comprehensive assessment Considers the synergy, toxic level and ecological sensitivity of heavy metals Widely used Precise scale	No GB values Necessity for toxic response values (however, only for Hg, Cd, As, Cu, Pb, Cr, Zn and Ni values are given) Ranking system of $E_{r}^{i}$ is required	Al-Anbari et al. (2015), Gong et al. (2008), Guan et al. (2014), Inengite et al. (2015), Ogunkunle and Fatoba (2013), Pejman et al. (2015), Sayadi et al. (2015), Tang et al. (2015), Wang et al. (2015) and Zhiyuan et al. (2011)
mCd	Assessment of overall degree of contamination	Easy to apply Indicates heavy metal as well as organic pollution Without the resistance of an upper limit Taking into account all analyzed heavy metals Widely used	Does not require variation of natural processes Omits the available ability of heavy metals Does not include GB	Abrahim and Parker (2008) and Nikolaidis et al. (2010)
ExF	Determination of the most polluted site point Overall soil assessment	Easy to apply Combines all heavy metals into one index	Not much used in the literature Does not allow the recognition of accumulation from natural contamination No precise scale	Bąbelewska (2010) and Sutherland (2000)

*I*_*geo*_ Geoaccumulation Index, *PI* Single Pollution Index, *EF* enrichment factor, *C*_*f*_ contamination factor, *BGI* Biogeochemical Index, *PI*_*sum*_ sum of contamination, *PI*_*Nemerow*_ Nemerow Pollution Index, *PLI* Pollution Load Index, *PI*_*avg*_ Average Single Pollution Index, *PI*_*Vector*_ Vector Modulus of Pollution Index, *PIN* background enrichment factor, *MEC* multi-element contamination, *CSI* Contamination Security Index, *MERMQ* the probability of toxicity, *C*_deg_ degree of contamination, *RI* potential ecological risk, *mCd* modified degree of contamination, *ExF* exposure factor, *GB* geochemical background

Calculation of soil pollution indices requires the assessment of the geochemical background (GB). This term was introduced to distinguish natural concentrations of heavy metals in the soil from abnormal concentrations (Reimann and Garret 2005). Many definitions have been used to characterize GB. Hawkes and Webb (1962) first defined GB as ‘the normal abundance of an element in barren earth material.’ According to Matschullat et al. (2000), GB ‘is characterized by spatio-temporal changes of the content of heavy metal, which is characteristic for the soil type or region’ and ‘reflects natural composition of heavy metals.’ Another definition of GB is given by Matschullat et al. (2000) as a ‘relative measure to distinguish between natural elements or compound concentrations and anthropogenically influenced concentration in real sample collections.’ Adamu and Nganje (2010) and Karim et al. (2015) concluded that GB ‘is a relative measure to differentiate between natural element or compound concentrations and anthropogenically-influenced concentrations in a given environmental sample.’ Reimann and Garret (2005) defined GB as ‘typical (normal, average) element concentrations in a specific area’ and ‘the concentration of a substance in a sample material at the distance to a source where the concentration of the substance can no longer be proven to originate from this source.’ According to Gałuszka (2007), GB ‘is a theoretical natural concentration range of a substance in a specific environmental sample (or medium), considering its spatial and temporal variability.’ Gałuszka and Migaszewski (2011) presented two approaches. Their first approach focuses on the difference between normal and anomalous contents of heavy metals in soil, water, etc., including the following definition: ‘GB is the normal concentration of a given element in a material under investigation, such as rock, soil, plants and water’ (Bates and Jackson 1984), encompassing the above-mentioned definition of Hawkes and Webb (1962). Their second approach is adequate for the definitions of GB mentioned before and given by Matschullat et al. (2000). A similar approach was mentioned by Dung et al. (2013).

GB used for the calculation of pollution indices should not be higher than the threshold, indicating the upper limit of the normal content of heavy metal concentrations in the soil (Reimann and Garret 2005). The term ‘threshold’ can also be defined as an ‘outer limit of background variation’ (Garrett 1991). GB definitions are connected with the term ‘baseline value.’ The baseline is the ‘present concentration of a chemical substance in a contemporary environmental sample’ (Garrett 1991) and ‘content of measuring levels “now” so that future change can be quantified’ (Reimann and Garret 2005).

Two kinds of GB were distinguished by Kowalska et al. (2016): reference and local (natural). The average content of heavy metals given in the literature, which can vary greatly due to localization differences and soil type, could be considered the reference geochemical background (RGB). In some regions of the world, geological magnetic anomalies occur, which should be taken into account during the selection of a GB (Chen et al. 2016; Lis and Pasieczna 1997; Xu et al. 2015; Zhou et al. 2015). An expression of these anomalies is a higher content of heavy metals in soils affected by the occurrence of nonferrous metal ores and by climatic factors (Pająk et al. 2015, 2017; Reimann and Garret 2005; Zhou et al. 2015). A different approach uses the local geochemical background (LGB) in the calculation of pollution indices. LGB is the concentration of heavy metals conditioned by natural processes characteristic of a particular area. Soil material is considered the LGB when it is not affected by human activity (Abrahim and Parker 2008; Reimann and Garret 2005).

So far, there has not been a comprehensive study related to the description of a wide spectrum of pollution indices including an indication of their strengths and weaknesses. The main objectives of this study are to: (1) present an exhaustive way to evaluate the ecological toxicology of heavy metals; (2) classify and compare the possibility of soil pollution assessment with the support of 18 relative indices given in the contemporary literature; (3) attempt to characterize the usefulness of indices according to land type use (farmland, forest and urban areas); and (4) solve the issue of choosing the appropriative GB.

## Methods

This review paper contains a comprehensive comparison of eighteen different indices of pollution chosen from the literature after an in-depth literature survey (Table 1). Equations for each of the described pollution indices and their suggested interpretations are also provided.

The reviewed pollution indices are divided into two groups: individual and complex. The first group contains indices that are calculated for each individual heavy metal separately. Complex pollution indices describe contamination of soil in a more holistic way, considering the content of more than one heavy metal or a sum of individual indices. Furthermore, in order to simplify the choice of appropriate indices, we have divided the pollution indices in terms of purpose and method of calculation (see Discussion section).

In order to show the similarities or differences between pollution indices, these were calculated based on the content of Cd, Pb and Zn given in the literature from 84 soils, representing different types of land use: farmland, forest and urban areas (Table 2). As a reference element, Sc content in soil given by Kabata-Pendias (2011) was used, which is necessary to calculate the enrichment factor (EF). For the calculation of BGI, the content of heavy metals in O and A horizons in forest soils was used. Calculations of pollution indices were conducted using heavy metal composition from the upper continental crust (UCC) proposed by Rudnick and Gao (2003) (Table 3). UCC constitutes an RGB that represents the lithogenic contents of heavy metals which are not under the influence of pedogenic processes. In this investigation, the reference (UCC) values and pollution indices provide a more universal character.

**Table 2:** References used to calculate analyzed pollution indices

Author(s)	Location	Use	Numbers of profiles
Pan et al. (2016)	China	Farmland	1
Inboonchuay et al. (2016)	N Thailand	Farmland	1
Wei and Yang (2010)	China	Farmland	1
Gutierrez et al. (2016)	Spain	Farmland	1
Valladares et al. (2009)	Brazil	Farmland	1
Rodríguez et al. (2013)	Spain	Farmland	1
Redon et al. (2013)	France	Farmland	2
Gu et al. (2014)	China	Farmland	1
Hajduk et al. (2012)	E Poland	Farmland	6
Obiora et al. (2016)	Nigeria	Farmland	3
Hovmand et al. (2008)	S Scandinavia	Forest	1
Pająk et al. (2015)	Poland	Forest	10
Karczewska and Kabała (2002)	S Poland	Forest	4
Ekwere et al. (2014)	Nigeria	Urban area	4
Xia et al. (2011)	China	Urban area	6
Markiewicz-Patkowska et al. (2005)	UK	Urban area	1
Wei and Yang (2010)	China	Urban area	1
Stajic et al. (2016)	Serbia	Urban area	14
Salah et al. (2015)	Iraq	Urban area	20
Liu et al. (2016)	Beijing	Urban area	1
Mahmoudabadi et al. (2015)	Iran	Urban area	1
Wu et al. (2015)	China	Urban area	1
Nannoni and Protano (2016)	Siena City	Urban area	2

**Table 3:** Geochemical backgrounds given in the literature and tolerable limits of heavy metals

Element	K-P (mg kg^−1^)	UCC	LCC	K
Ag	0.13	53	50	–
As	0.67	4.8	1.6	20
Cd	0.41	0.09	0.098	3
Cr	59.5	92	85	–
Cu	38.9	28	25	100
Ga	15.2	17.5	17	–
Hg	0.07	0.05	–	2
Mn	488	438.59	–	–
Ni	29	47	44	100
Pb	27	17	17	100
Sn	2.5	2.1	5.5	–
Zn	70	67	71	300

*K-P* Kabata-Pendias (2011), average content in surface horizons worldwide, *UCC* Rudnick and Gao (2003), composition in upper continental crust, *LCC* McLennan (2001), composition in lower continental crust, *K* Kloke (1979), tolerable levels in soils

To aid in the determination of relationships between pollution indices, Ward’s hierarchical cluster analysis (HCA) method as well as principal component analysis (PCA) were applied using Statistica^®^ version 12.0 software. HCA is considered a practical way to gather a variety of data sets by creating groups. This clustering is based on the agglomeration method that estimates linkage distance. In this case, the estimation of differences between particular groups takes place (Murtagh and Legendre 2014). HCA depends on organizing all the data in the structure in such a way that the degree of linkage of the objects (indices) belonging to the same cluster is the greatest. In this study, data are presented as dendrograms (tree diagrams). The principles of dendrogram interpretation involve graphical analysis. This method results in the collation of an increasing number of indices on the basis of their characteristics (Murtagh and Legendre 2014). In turn, PCA is widely used as a way to identify patterns within a set of data (Rao 1964; Smith 2002; Wold et al. 1987; Zhiyuan et al. 2011). Presentation of data by PCA aims to highlight their similarities and differences (Smith 2002). Often, PCA is used when graphical presentation of data is not available. The PCA model is based on total variance. The main advantage of PCA consists in data compression by reducing the large number of variables to a small set, which nonetheless still contains most of the information across a wide range (Rao 1964; Wold et al. 1987). With PCA, unities are used in the diagonal of the correlation matrix computationally implying that the variance is common (Smith 2002). In general, the interpretation of PCA is based on gathering all the similarities in one quarter: the closer the distance between components, the more the similarities that can be found between them (Gąsiorek et al. 2017; Zhiyuan et al. 2011). Further, PCA is useful for the comparison of patterns between studied pollution indices and the establishing of possible similarities (Chen et al. 2015; Varol 2011; Zhiyuan et al. 2011). Moreover, PCA allows the assessment of overall variability across the pollution indices.

In our study, PCA diagrams were drawn up for individual and complex pollution indices separately. Such a method of division was supported by specific values/numbers, e.g., GB, using for every pollution indice calculation. Due to the limited space for figures, we decided to show only those PCA diagrams where positive correlations were found between indices.

## Pollution indices

### Individual indices

The individual indices group contains tools that can be used for the unitary assessment of soil pollution with particular heavy metals. Besides the content of heavy metals in soil, knowledge of the GB or other reference data obtained from the literature may be necessary.

#### Geoaccumulation Index *(I*_geo_)

*I*_geo_ allows the assessment of soil contamination with heavy metal based on its contents in A or O horizons referenced to a specified GB (Müller 1969).

1 $$I_{\text{geo}} = \log_{2} \left[ {\frac{\text{Cn}}{{1.5 \,{\text{GB}}}}} \right]$$

where Cn—concentration of individual heavy metal, GB—value of geochemical background and 1.5—constant, allowing for an analysis of the variability of heavy metals as a result of natural processes.

*I*_geo_ values are helpful to divide soil into quality classes (Müller 1969; Nowrouzi and Pourhabbaz 2014). Please see Table S1 (Supplementary material) for interpretation of results.

#### Single Pollution Index (PI)

An index that can be used to determine which heavy metal represents the highest threat for a soil environment is the Single Pollution Index (PI). This is also necessary for the calculations of some of complex indices, e.g., the Nemerow Pollution Index (PI_Nemerow_) (Guan et al. 2014) and the Pollution Load Index (PLI) (Varol 2011), and is described below.

2 $${\text{PI}} = \frac{{\text{Cn}}}{{\text{GB}}}$$

where Cn—the content of heavy metal in soil and GB—values of the geochemical background.

Table S2 presents an interpretation of the PI values.

#### Enrichment factor (EF)

EF is a measure of the possible impact of anthropogenic activity on the concentration of heavy metals in soil. To identify the expected impact of anthropogenesis on the heavy metal concentrations in the soil, the content of heavy metals characterized by low variability of occurrence (LV) is used as a reference, both in the analyzed samples and in GB. Reference elements are usually Fe, Al, Ca, Ti, Sc or Mn. *EF* is calculated using the following formula (Sutherland 2000):

3 $${\text{EF}} = \frac{{\left[ {\frac{\text{Cn}}{{\rm LV}} } \right]\,{\text{sample}}}}{{\left[ {\frac{\text{GB}}{{\rm LV }}} \right]\,{\text{background}}}}$$

where $\left[ {\frac{\text{Cn}}{{\rm LV}} } \right]\,{\text{sample}}$—content of analyzed heavy metal (Cn) and one of the following metals Fe/Al/Ca/Ti/Sc/Mn (LV) in the sample and $\left[ {\frac{\text{Cn}}{{\rm LV}} } \right]\,{\text{background}}$—reference content of the analyzed heavy metal (Cn) and one of the following metals Fe/Al/Ca/Ti/Sc/Mn (LV).

If the value of EF ranges from 0.5 to 1.5 (Table S3), it can be stated that the content of that particular heavy metal in the soil is caused by natural processes. However, if the value of EF exceeds 1.5, there is a possibility that the heavy metal contamination occurred as a result of anthropogenic activities (Elias and Gbadegesin 2011; Zhang and Liu 2002).

#### Contamination factor (*C*_f_*,)*

The assessment of soil contamination can also be carried out using *C*_f_. This index enables the assessment of soil contamination, taking into account the content of heavy metal from the surface of the soil and values of pre-industrial reference levels given by Håkanson (1980) (Table S4).

*C*_f_ is calculated by the following formula:

4 $$C_{\text{f}} = \frac{\text{Cm}}{{C_{{{\text{p}} - {\text{i}}}} }}$$

where Cm—mean content of heavy metal from at least five samples of individual metals and *C*_p−i_—pre-industrial reference value for the substances (Table S4).

Table S5 provides an interpretation of *C*_f_ values.

### A newly introduced index: the Biogeochemical Index (BGI)

There is no universal index in the literature to evaluate the degree of heavy metal concentration in the O horizon of soils under forest and grassland vegetation. The Biogeochemical Index (BGI) (Mazurek et al. 2017) is designed to fill this gap. For the calculations, knowledge of the heavy metal content in the O horizon and the directly underlying A horizon is necessary. It can be assumed that the higher the BGI values, the greater the capability of the O horizon to sorb heavy metals and neutralize xenobiotics, as well as reduce phytotoxicity. BGI is calculated by:

5 $${\text{BGI}} = \frac{{C_{n} {\text{O}}}}{{C_{n} {\text{A}}}}$$

where *C*_*n*_O—content of a heavy metal in the O horizon and *C*_*n*_A—content of a heavy metal in the A horizon.

Interpretations of BGI are suggested in Table S6. BGI is helpful to determine the ability of the O horizon to sorb pollutants. Thus, values above 1.0 demonstrate increased ability of heavy metal sorption by the O horizons of soil. However, one should take into account the fact that the index does not consider the density of soil particles of O and A horizons; hence, BGI is only an approximation (Mazurek et al. 2017).

### Complex indices

The complex indices group allows the specification, in a comprehensive way, of the degree of heavy metal pollution. For the calculation of each of the complex indices, total concentrations of all analyzed heavy metals in soils as well as (in some cases) individual values of the calculated indices were used.

#### Sum of contamination (PI_sum_)

A commonly applied index of heavy metal contamination in soils is the sum of contamination (PI_sum_). It can be defined as the sum of all determined contents of heavy metals in the soil, expressed as PI (Gong et al. 2008). It is calculated using the formula:

6 $${\text{PI}}_{\text{sum}} = \mathop \sum \limits_{i - 1}^{n} {\text{PI}}$$

where PI—calculated values for Single Pollution Index and *n*—the number of total heavy metals analyzed in this study.

#### Nemerow Pollution Index (PI_Nemerow_)

The Nemerow Pollution Index (PI_Nemerow_) allows the assessment of the overall degree of pollution of the soil and includes the contents of all analyzed heavy metals (Gong et al. 2008). It is calculated for both the O and A horizons, based on the following formula:

7 $${\text{PI}}_{\text{Nemerow}} = \sqrt {\frac{{\left( {\frac{1}{n}\mathop \sum \nolimits_{i - 1}^{n} {\text{PI}}} \right)^{2} + {\text{PI}}_{{ {\text{max}}}}^{2} }}{n}}$$

where PI—calculated values for the Single Pollution Index, PI _max_—maximum value for the Single Pollution Index of all heavy metals and *n*—the number of heavy metals.

Based on PI_Nemerow_, five classes of soil quality were created (Table S7).

#### Pollution Load Index (PLI)

For the total assessment of the degree of contamination in soil, the PLI is also used. This index provides an easy way to prove the deterioration of the soil conditions as a result of the accumulation of heavy metals (Varol 2011). PLI is calculated as a geometric average of PI based on the following formula:

8 $${\text{PLI}} = \sqrt[n]{{{\text{PI}}_{1} \times {\text{PI}}_{2} \times {\text{PI}}_{3} \times \ldots .{\text{PI}}_{n} }}$$

where *n*—the number of analyzed heavy metals and PI—calculated values for the Single Pollution Index.

PLI classes are shown in Table S8.

#### Average Single Pollution Index (PI_avg_)

PI_avg_ was first used by Gong et al. (2008) and Inengite et al. (2015) in order to estimate soil quality. It can be defined as follows:

9 $${\text{PI}}_{\text{avg}} = \frac{1}{n} \mathop \sum \limits_{i = 1}^{n} {\text{PI}}$$

where *n*—the number of studied heavy metals and PI—calculated values for the Single Pollution Index.

PI_avg_ values in excess of 1.0 show a lower quality of the soil, which is conditioned by high contamination (Inengite et al. 2015).

#### Vector Modulus of Pollution Index (PI_Vector_)

This index was introduced by Gong et al. (2008) and is defined as:

10 $${\text{PI}}_{\text{Vector}} = \sqrt {\frac{1}{n}} \mathop \sum \limits_{i = 1}^{n} {\text{PI}}^{2}$$

where *n*—the number of determined heavy metals and PI—calculated values for the Single Pollution Index.

#### Background enrichment factor (PIN)

Introduced by Caeiro et al. (2005), PIN is helpful to assess the enrichment of soil by heavy metals using class contamination of PI (Table S2) as well as appropriate GB values. PIN is defined as:

11 $${\text{PIN}} = \mathop \sum \limits_{i = 1}^{n} \frac{{{\text{PIClass}}^{2} \times {\text{Cn}}}}{\text{GB}}$$

where PIClass—class of heavy metal pollution (given in Table S2), Cn—contamination by heavy metal and GB—geochemical background.

An interpretation of PIN values is given in Table S9.

#### Multi-element contamination (MEC)

Using MEC gives a measure to assess contamination based on the content of heavy metals in surface soil horizons, with the limits given by Kloke (1979) (Table 3). This index was introduced by Adamu and Nganje (2010). MEC values above 1.0 testify to an anthropogenic impact on heavy metal concentration in soil. MEC is calculated based on the following formula:

12 $${\text{MEC}} = \frac{{\left( {\frac{{C_{1} }}{{T_{1} }} + \frac{{C_{2} }}{{T_{2} }} + \frac{{C_{3} }}{{T_{3} }} + \ldots \frac{{C_{n} }}{{T_{n} }}} \right)}}{n}$$

where *C*—content of heavy metal, *T*—tolerable levels given by Kloke (1979) (Table 3) and *n*—the number of heavy metals.

#### Contamination Security Index (CSI)

CSI is informative in terms of the concentration of heavy metals in the soil. It was introduced by Pejman et al. (2015). In order to calculate CSI, ‘effects range low’ (ERL) and ‘effects range median’ (ERM) values given by Long et al. (1995) (presented in Table S10) are used. CSI is also helpful to determine the limit of toxicity above which adverse impacts on the soil environment are observed. The index is calculated according to the formula:

13 $${\text{CSI}} = \mathop \sum \limits_{i = 1}^{n} w\left( {\left( {\frac{C}{\text{ERL}}} \right)^{{\frac{1}{2}}} + \left( {\frac{C}{\text{ERM}}} \right)^{2} } \right)$$

where *W*—computed weight of each heavy metal according to Pejman et al. (2015) (Table S11), *C*—concentration of heavy metal, and ERL, ERM—values from Table S10.

An interpretation of *CSI* values is given in Table S12.

#### The probability of toxicity (MERMQ)

This index is used as an instrument to recognize the harmful impact on the soil environment of heavy metals (Gao and Chen 2012; Pejman et al. 2015). MERMQ is calculated based on the following formula:

14 $${\text{MERMQ}} = \frac{{\mathop \sum \nolimits_{i = 1}^{n} \frac{{\text{Cn}}}{{\text{ERM}}}}}{n}$$

where *Cn*—concentration of each analyzed heavy metal, ERM—values given by Long et al. (1995) (Table S10) and *n*—the number of analyzed heavy metals.

An interpretation of this index is shown in Table S13.

#### Degree of contamination (*C*_deg_*,)*

According to Håkanson (1980), the assessment of contamination can be carried out by using the degree of contamination index, *C*_deg_, which is calculated as follows:

15 $$C_{{\rm deg}} = \mathop \sum \limits_{i = 1}^{n} C_{\text{f}}$$

where *C*_f_—contamination factor and *n*—the number of analyzed heavy metals.

An interpretation of *C*_deg_ is shown in Table S5.

#### Potential ecological risk (RI)

Potential ecological risk (RI) is an index applicable for the assessment of the degree of ecological risk caused by heavy metal concentrations in the water, air, as well as the soil. This index was introduced by Håkanson (1980), and it is calculated using the following formula:

16 $${\text{RI}} = \mathop \sum \limits_{i = 1}^{n} E_{\text{r}}^{i}$$

where *n*—the number of heavy metals and *E*_r_—single index of the ecological risk factor calculated based on the equation:

17 $$E_{\text{r}}^{i} = T_{\text{r}}^{i} \times {\text{PI}}$$

where *T*_r_^*i*^—the toxicity response coefficient of an individual metal (Håkanson 1980) (Table S4) and PI—calculated values for the Single Pollution Index.

Based on the potential ecological risk, five classes of soil quality were distinguished (Table S14).

#### Modified degree of contamination (mCd)

This index was first used by Abrahin and Parker (2008). It allows the assessment of the overall heavy metal soil contamination. To calculate this index, the sum of the content of heavy metals is necessary. An interpretation of *mCd* values is shown in Table S15. mCd is calculated using the following formula:

18 $${\text{mCd}} = \frac{{\mathop \sum \nolimits_{i = 1}^{n} {\text{Cn}}}}{n}$$

where *n*—the number of analyzed heavy metals and Cn—content of individual heavy metal.

#### Exposure factor (ExF)

ExF is very useful to assess where, in a given study area, the greatest heavy metal loads are located (Bąbelewska 2010), and it is calculated as follows:

19 $$y = \mathop \sum \nolimits \frac{{{\text{Cn}} - C_{\text{av}} }}{{C_{\text{av}} }}$$

where Cn—content of heavy metal at an analyzed sampling point and *C*_av_—average content of heavy metal in the soil profile.

## Discussion

Characterization of indices based on their scope, method of calculation as well as strengths and weaknesses

The contemporary approach presupposes that the simultaneous use of several indices has been found to more accurately assess heavy metal pollution in soil (Table 1). Several studies have reported that the selection of pollution index is connected with different aims, such as contamination level, heavy metal origin or ecological potential risk (Al-Anbari et al. 2015; Baran et al. 2018; Dung et al. 2013; Guan et al. 2014; Obiora et al. 2010; Qingjie et al. 2008).

Pollution indices can be divided into six groups based on the different purposes of calculation, i.e., to provide information about: (1) individual levels of pollution from each of the analyzed heavy metals (*I*_geo_, PI*, C*_f_); (2) the scale of total pollution (PI_sum_, PI_Nemerow_, PLI, PI_ave_, mCd, PI_Vector_*, C*_deg_, PIN and SCI); (3) the source of heavy metals (EF and MEC); (4) the potential ecological risk (RI and MERMQ); (5) the area with the highest potential risk of heavy metal accumulation (ExF); and (6) the ability of the surface horizon to accumulate heavy metals (BGI).

**Fig. 1:**
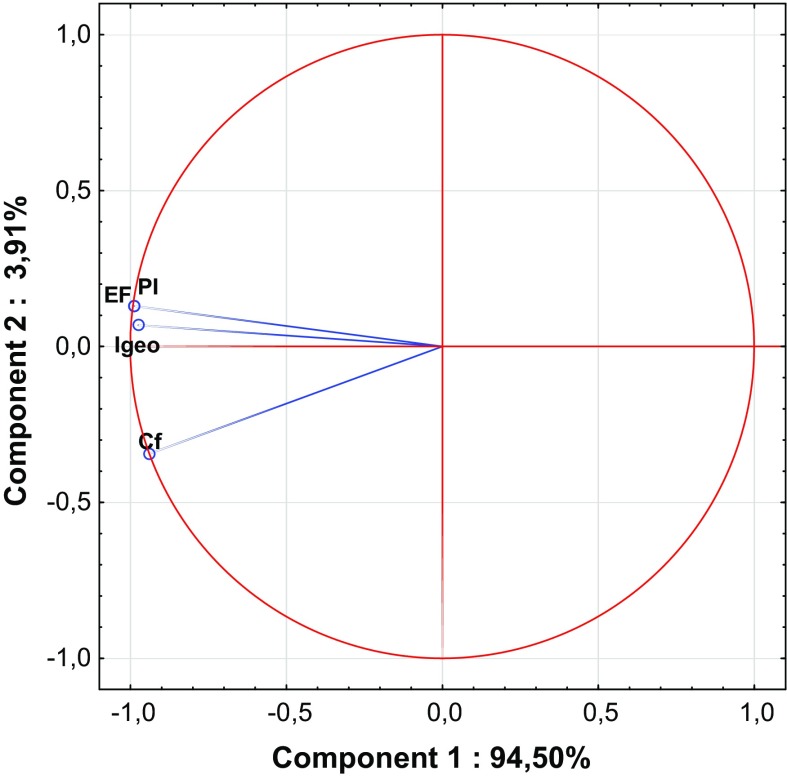
Principal component analysis (PCA) biplot for the individual indices

**Fig. 2:**
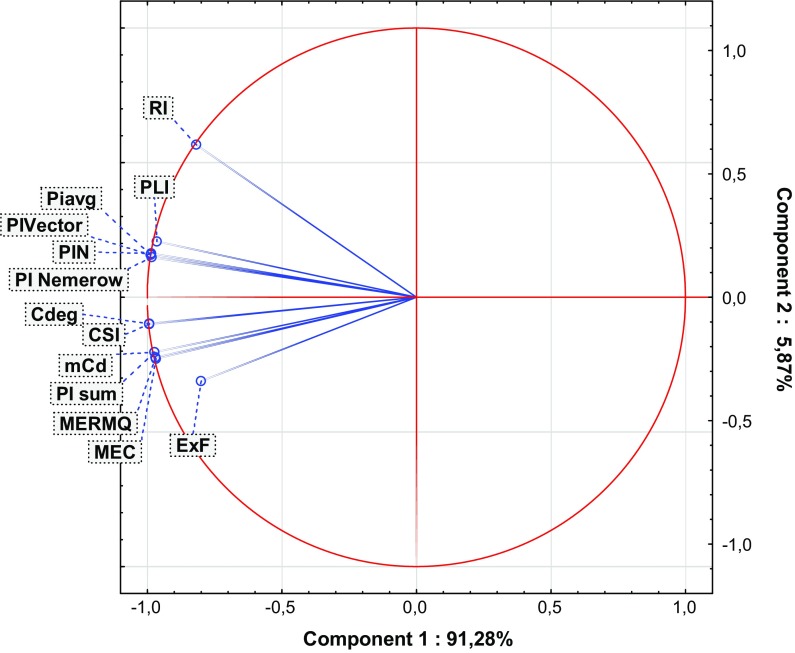
Principal component analysis (PCA) biplot for the complex indices

Ward’s hierarchical cluster analysis (HCA), as well as principal component analysis (PCA), is helpful to standardize pollution indices to allow better comparison between them (Wang et al. 2015; Qingjie et al. 2008; Wold et al. 1987; Zhiyuan et al. 2011). The listed pollution indices have a lot of common attributes. Similarly, PCA showed that variation between indices is based mainly on the measurement of the health and quality of the soil (Figs. 1 and 2, Table 4). The clearest similarities result from the calculation methods (Dung et al. 2013; Guan et al. 2014; Inengite et al. 2015). Across the individual and complex pollution indices descriptive statistics were used: geometric sum, geometric average or weighted geometric values. Furthermore, some of the pollution indices are based on reference data, such as GB, or use other specified values which differ from traditional GB (Gao and Chen 2012; Wang et al. 2015). Hence, taking into consideration the method of calculation, pollution indices can be divided into three groups: (1) indices that are based on the calculation of GB values (EF*, I*_geo_, PI, PI_sum_, PI_Nemerow_, PI_avg_, PI_Vector_, PIN and PLI); (2) indices that are calculated based on data other than GB given in the literature (*C*_f_, MEC, C_deg_, RI, MERMQ and CSI); and (3) indices that are calculated based on heavy metal content in the analyzed soil profile but not in parent material (BGI, mCd and ExF).

**Table 4:** Principal component loadings for complex index values

Index	PCA1	PCA2
PI_sum_	− 0.973	− 0.204
PI_Nemerow_	− 0.983	0.148
PLI	− 0.964	0.207
PI_avg_	− 0.987	0.157
PI_Vector_	− 0.987	0.157
PIN	− 0.986	0.162
MEC	− 0.967	− 0.228
CSI	− 0.992	− 0.100
MERMQ	− 0.969	− 0.223
*C*_deg_	− 0.992	− 0.097
RI	− 0.817	0.565
mCd	− 0.973	− 0.204
ExF	− 0.799	− 0.311

**Fig. 3:**
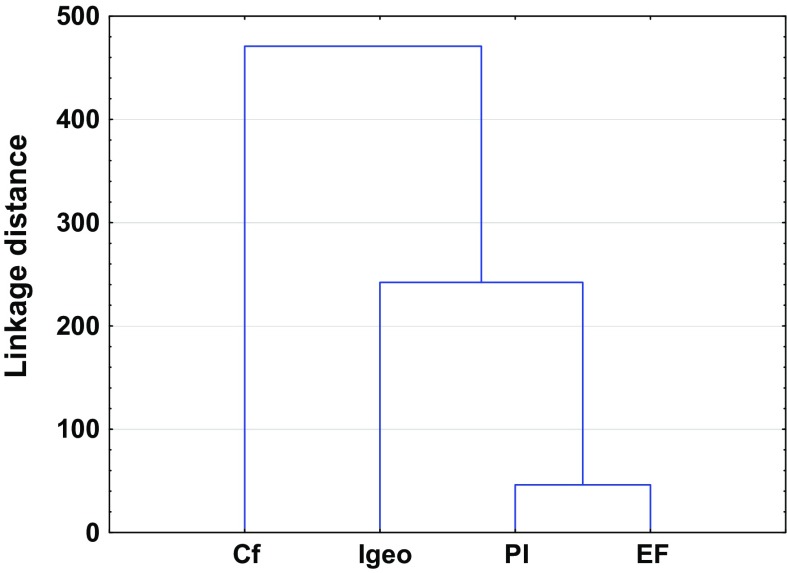
Ward’s hierarchical cluster analysis of the studied individual pollution indices based on different land uses

**Fig. 4:**
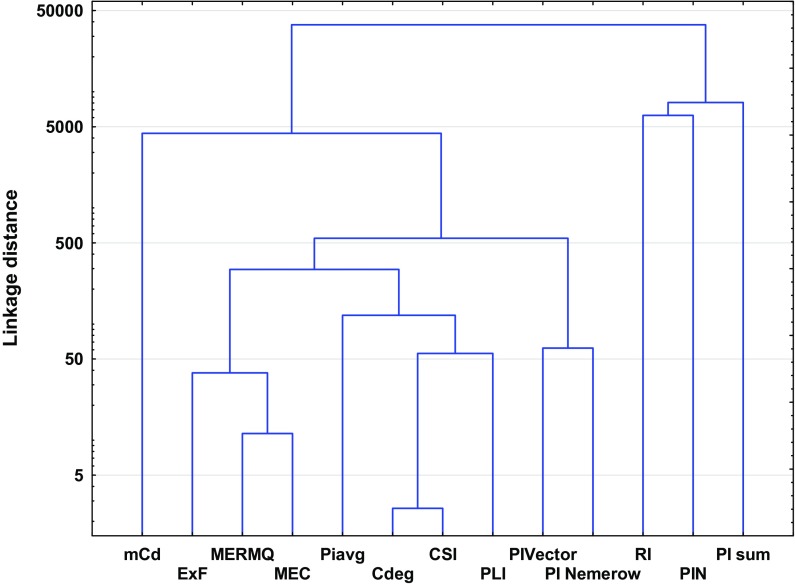
Ward’s hierarchical cluster analysis of the studied complex pollution indices based on different land use

Despite the obvious similarities, the pollution indices differ from each other due to various factors that affect their importance (Kowalska et al. 2016). Thus, some of the studied pollution indices may not be readily comparable (Dung et al. 2013; Gao and Chen 2012). These differences are confirmed statistically by high linkage distances between clusters (Figs. 3 and 4). Theoretically, the higher the linkage distance, the more diverse the traits the two indices have. Hence, analogously, the lower the linkage distances, the fewer the differences between indices (Murtagh and Legendre 2014). The discussion below and also Table 1 highlight the advantages and disadvantages of individual and complex pollution indices.

From among the individual pollution indices, *I*_geo_ and PI are considered to be the most accurate and have been used for a few decades to evaluate the degree of contamination (Table 1) (Begum et al. 2014; Karim et al. 2015; Li and Yang 2008; Sayadi et al. 2015). Those indices allow the comparison of previous and present contamination, which they treat in quite similar ways. *I*_geo_ and likewise PI should be calculated with respect to appropriate GB. Thus, the key for appropriate calculation is the choice of GB (Kowalska et al. 2016; Matschullat et al. 2000). On the other hand, neither of these indices includes the variation of natural processes. One of the biggest disadvantages for the above-mentioned factors is the lack of consideration of the impact of heavy metals on edaphic properties and xenobiont behavior in the soil (Dung et al. 2013; Jiang et al. 2014; Mmolawa et al. 2011; Sayadi et al. 2015). Nevertheless, these indices are characterized by their very precise scale. Only *I*_geo_ allows minimization of the degree of accumulation resulting from artificial footprints of human activity (by the 1.5 factor), which offers a significant advantage over other individual indices (Li et al. 2016). Similarities between these two indices are clearly visible in the PCA diagram (Fig. 1).

An index based on differentiation between anthropogenic and natural pollution sources is EF (Dung et al. 2013; Kowalska et al. 2016; Reimann and De Caritat 2005). Calculation of EF is connected with the standardization of element measures (Reimann and De Caritat 2005). EF is the only one of the studied indices that includes low occurrence of variability elements (Abrahim and Parker 2008; Bourennane et al. 2010; Karim et al. 2015). EF, similar to *I*_geo_ and PI, is a tool that involves the geochemical values. In the calculation of this index, RGB values have very often been used. Some authors have claimed that LGB should be taken into account as well (Kowalska et al. 2016). For the calculation of EF, it is necessary to know the level of enrichment of the sample and the reference values, which are often characterized by low occurrence variability (Omatoso and Ojo 2015). This measure is used in order to normalize the geochemical influence and differentiate between heavy metals originating from human activities and those of natural sources (Reimann and De Caritat 2005; Mmolawa et al. 2011; Sutherland 2000). The choice and determination of the element demonstrating low levels of variability (Table 1) should be connected with the type and properties of the studied soil, which may sometimes bring some uncertainty, and this may be one of the only drawbacks of this index. The fifth grade of the EF scale (Table S3) allows easy detection of anthropogenic influences (Gąsiorek et al. 2017; Reimann and De Caritat 2005; Wang et al. 2015; Varol 2011).

Distinct from other individual indices is *C*_f_, a fact which has also been confirmed statistically (Figs. 1, 3). No GB data are needed in *C*_f_ calculations (Abrahim and Parker 2008; Li et al. 2016; Loska et al. 2004; Varol 2011). Nevertheless, this index focuses on the ratio between actual contamination by an individual heavy metal and pre-industrial reference data given by Håkanson (1980) (Table S4). Such an approach excludes the possibility of the inappropriate choice of GB, thereby reducing inconsistencies in the obtained pollution index values. It should be noted that *C*_f_ also does not include the variation of natural processes (Dung et al. 2013; Loska et al. 2004; Varol 2011).

Another somewhat ‘separated’ pollution index is BGI. This index, similar to PI, is based on the ratio between contamination of different horizons/layers. It is important for highlighting pollution levels in forested areas, as has been confirmed by Mazurek et al.’s (2017) study. BGI needs to be calculated to characterize the natural buffering properties of the O horizon, which provides biosorption of contaminants (De Santo et al. 2002; Pająk et al. 2015). Moreover, BGI has the potential to show the vertical mobility of heavy metals (Mazurek et al. 2017).

The tools used for overall soil pollution evaluation are the complex indices (Table 1). Complex pollution indices integrate and average all available analytical data (Abrahim and Parker 2008; Dung et al. 2013). Some of these provide complementary information and allow comparison of the degree of overall contamination in different sites due to the use of a specific, common scale (Qingjie et al. 2008). Among the complex pollution indices, a series of similar indices can be distinguished, i.e., PI_sum_, PI_Nemerow_, PLI, PI_avg_, PI_Vector_ and PIN. The similarities between these indices manifest themselves in their similar purposes (Inengite et al. 2015), calculations with regard to *PI* values, and are readily comparable due to their nature (Gong et al. 2008; Inengite et al. 2015). The above-mentioned similarities are confirmed by PCA scatter plots (Fig. 2, Table 4). All ‘*PI*-indices’ are calculated (indirectly) with respect to GB (Gałuszka and Migaszewski 2011). Moreover, these pollution indices are characterized by their simplicity of application, can be easily understood and interpreted, and also show acceptable levels of contamination (Caeiro et al. 2005; Inengite et al. 2015; Shu and Zhai 2014). Some ‘*PI*-indices’ have no precise scale or are ‘single-scaled’ (PI_sum_, PI_Vector_ and PI_avg_, respectively), which represents a disadvantage in some cases. ‘*PI*-indices’ depend on PI values, which are strictly connected with GB and may often lead to some shortcomings where the wrong choice of GB has been made (Kowalska et al. 2016; Mazurek et al. 2017).

MEC is a widely used tool for generating information about heavy metal origin (Adamu and Nganje 2010; Pejman et al. 2015). This may be comparable to the EF pollution index. MEC is quite a new index and thus has not been widely used (Adamu and Nganje 2010). MEC values are not dependent on GB; nevertheless, they are based on data given by Kloke (1979) (Table 3). The weaknesses of this index are their poor scale, which can provide less comprehensive information than other indices (Dung et al. 2013; Pejman et al. 2015). According to the results of statistical analyses (Figs. 2 and 4), MEC may be correlated with MERMQ, but this is only due to the similar method of calculation (Adamu and Nganje 2010; Pejman et al. 2015).

Other complex pollution indices which are not connected with conventional GB values are CSI, MERMQ and *C*_deg_ (Table 1). CSI and MERMQ are based on ERM (effects range median) and ERL (effects range low) values (Table S10) instead of GB (Gao and Chen 2012; Han et al. 2016; Pejman et al. 2015; Wang et al. 2015). ERM and ERL values have been determined based on numerous toxicity tests, field studies, and delineate concentration ranges for many elements (Gao and Chen 2012; Gąsiorek et al. 2017; Han et al. 2016; Long et al. 1995). These indices are able to provide spatially representative patterns of soil contamination (Pejman et al. 2015). Terminology used for these complex indices differs from each other due to assessments based on different grades (Table S12 and S13). CSI includes a computed weight for every heavy metal in terms of overall contamination, which confirms its accuracy (Pejman et al. 2015; Wang et al. 2015). In turn, MERMQ determines the percentage probability of toxicity and is useful to find harmful human effects, a key to recognition of exposure to pollution (Gao and Chen 2012; Pejman et al. 2015). *C*_deg_ is more reliable and appropriate for the determination of site-specific contamination (Håkanson 1980). However, this index is strictly dependent on *C*_f_ values. *C*_deg_ represents a straightforward method of calculation and simple interpretation (Abrahim and Parker 2008). The similarities between the above-mentioned indices have also been confirmed statistically, especially those between *C*_deg_ and CSI (Figs. 2, 4, Table 4).

Among the complex pollution indices, RI may be considered a guideline for recognition of potential ecological risk (Al-Anbari et al. 2015; Hong-gui et al. 2012; Håkanson 1980; Jiang et al. 2014; Obiora et al. 2016; Sayadi et al. 2015). RI is one of the first introduced and the best known pollution indices (Table 1). The interpretation of RI is essential for decision-making processes and management, including protection of natural resources, and considers toxic levels, ecological sensitivity and synergies between heavy metals (Caeiro et al. 2005; Gąsiorek et al. 2017; Mazurek et al. 2017). This index requires a specific toxicity response coefficient (Håkanson 1980). The toxicity response coefficient is equivalent to the different toxicity values of particular elements. RI is characterized by a high level of accuracy due to its precise scale (Table S14). A small disadvantage of RI is the fact that the toxicity response coefficient has not been determined for a wide range of heavy metals (Table 1). A lack of clear linkages between RI and other indices is apparent (Figs. 2, 4, Table 4), which may suggest the individuality of this index and its low similarity to other indices (Chen et al. 2015; Kowalska et al. 2016).

With regard to an overall measurement of heavy metals in the soil profile, and also their lack of any need for the use of GB, mCd and ExF were considered (Table 1). There is not much information about these indices in the literature (Abrahim and Parker 2008, Bąbelewska 2010; Pejman et al. 2015). ExF does not allow the differentiation of anthropogenic accumulation from natural contamination, and there is no clear threshold between polluted and unpolluted sites (Bąbelewska 2010; Nikolaidis et al. 2010; Sutherland, 2000). In contrast to the other indices, ExF is able to identify locations with the highest probability of the occurrence of contaminants (Bąbelewska 2010; Pejman et al. 2015). Differences for this index relative to others (e.g., *PI*-indices) are also found based on statistical analyses, i.e., correlation or regression (Figs. 2, 4, Table 4). Moreover, a strongly negative connection has been noted between variability principal components of ExF and other indices, which proves the association between the spatial arrangement of environmental risk and the variance between index values (Fig. 2). Both ExF and mCd allow the ranking of primary contaminants (Abrahim and Parker 2008; Bąbelewska 2010; Nikolaidis et al. 2010). The mCd index has an advantage over ExF due to the development of a more precise scale (Table S15).

Taking into account their strengths and weaknesses, some of the pollution indices may be recommended by the authors of this review as being the most useful. Among individual pollution indices, we note both *I*_geo_, which provides information concerning contamination level, as well as EF, on the basis of which the origin of heavy metals can be determined (Abrahim and Parker 2008; Kowalska et al. 2016; Mazurek et al. 2016). Among the complex pollution indices the most useful as well as most universal in character are CSI and RI (Gąsiorek et al. 2017; He 2015). CSI is helpful to assess the overall level of accumulation of heavy metals and further determines their intensity (He 2015; Ololade 2014). In turn, RI is important because of its ability to define ecological risk (Håkanson 1980; Gong et al. 2008; Kowalska et al. 2016).

Choice of useful pollution indices according to soil use (farmland, forest and urban areas).

**Fig. 5:**
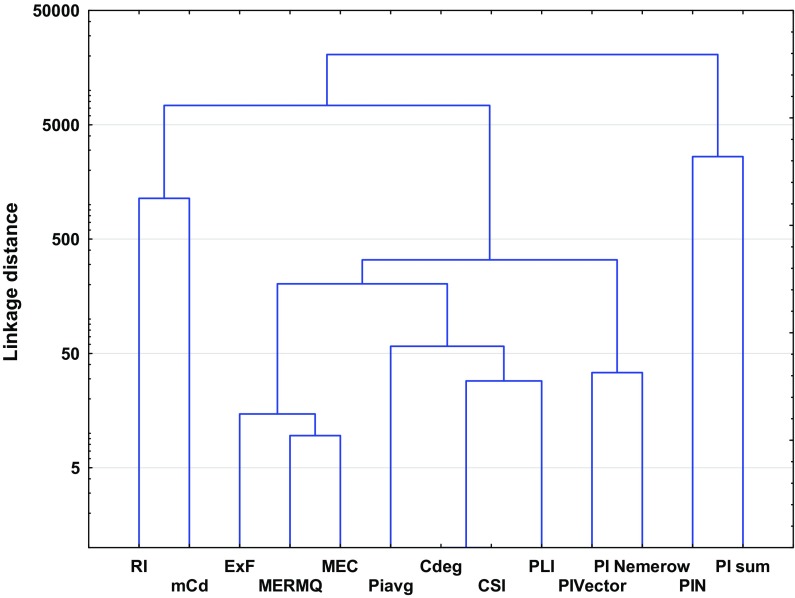
Ward’s hierarchical cluster analysis of the studied complex pollution indices based on farmland soils

The appropriateness of the various pollution indices differs depending on soil use (Gąsiorek et al. 2017; Mahmoudabadi et al. 2015; Mazurek et al. 2017; Ololade 2014; Wu et al. 2015). To understand the degree of pollution at a particular site, choice of appropriate indices is key and is based on both the risks resulting from their use as well as the purpose for which the pollution indices were developed (Begum et al. 2014; Chen et al. 2015; Gong et al. 2008; Kowalska et al. 2016). On farmland soils, understanding the degree of pollution is important for proper environmental management (Kelepertzis 2014; Kouamé et al. 2013; Rodríguez et al. 2013). Knowledge about soil pollution is important to reduce the risk of environmental exposure and to protect valuable ecosystems (Pan et al. 2016). Moreover, most agricultural practices (e.g., fertilizer and biocide application) contribute to overall heavy metal enrichment of soil and groundwater (Kouamé et al. 2013; Su et al. 2014). Hence, considerably disturbed soil can be attributed to multiple sources—natural and anthropogenic. We suggest that the most appropriate choice of individual pollution indices for farmland soils should include EF, as this will help identify the source of contamination (Kowalska et al. 2016). Further, it is important to use complex pollution indices which allow the determination of the potential ecological risk, as well as indication of the overall degree of pollution (Al-Anbari et al. 2015; Chen et al. 2015; Obiora et al. 2016). In the selection of appropriate pollution indices, cluster analysis may be helpful (Murtagh and Legendre 2014). Based on HCA, two main clusters are recognized (Fig. 5); the choice of index to assess the overall level of farmland soil pollution should include one of the ‘*PI*-indices,’ and one of the following indices: mCd, ExF, MERMQ, MEC, *C*_deg_ and CSI. RI does not exhibit similarity with the other complex pollution indices (Fig. 5), so its calculation is mandatory in the case of farmland soils.

Forest soil ecosystems require the assessment of heavy metal pollution within the surface O and A horizons (De Santo et al. 2002; Hovmand et al. 2008; Kaste et al. 2011; Karczewska and Kabała 2002; Kawahigashi et al. 2011; Mazurek et al. 2017). The pollution of organic matter with heavy metals could directly limit nutrient availability in soil (Kaste et al. 2011; Karczewska and Kabała 2002). The decomposition of organic matter may entail great changes in metal speciation over short timescales (Schroth et al. 2008). In the case of the composition of coniferous forest litter, it has been found that higher values for heavy metal content exist in needles compared with other organic components (Mazurek and Wieczorek 2007; Pająk et al. 2015). O horizons are able to bind large amounts of heavy metals from anthropogenic sources, which may be transferred over long distances and deposited on the surface horizon. These allogenic components contribute to changes in soil chemical composition (Kawahigashi et al. 2011; Pająk et al. 2017). Comprehensive assessment of heavy metal pollution within forest soils should include application of some complex indices.

**Fig. 6:**
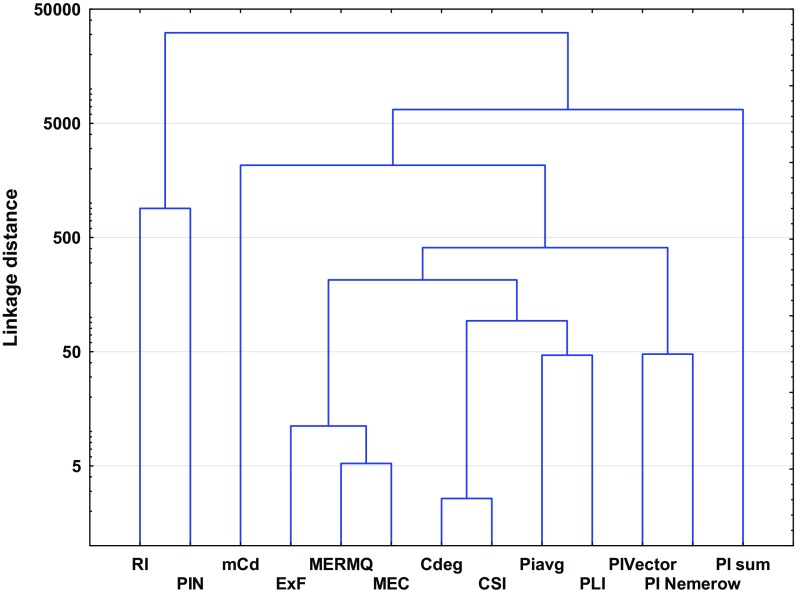
Ward’s hierarchical cluster analysis of the studied complex pollution indices based on forest soils

**Fig. 7:**
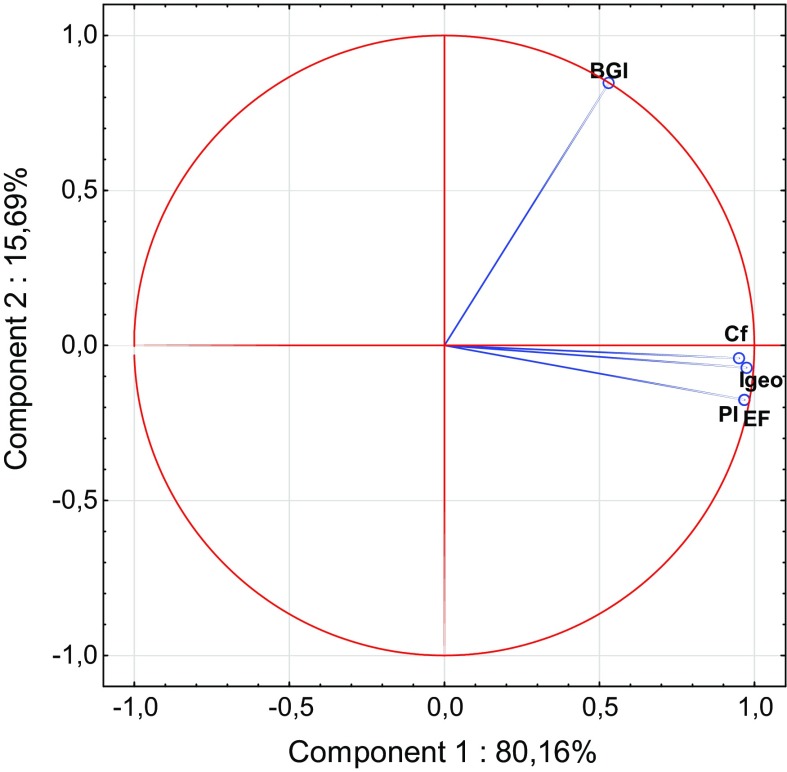
Principal component analysis (PCA) biplot for the individual indices computed for forest soils

Statistical analysis revealed that similar to the case of farmland soils, for overall assessment of contamination in forest soils, application of one of the ‘*PI*-indices’ is required or mCd, ExF, MERMQ, MEC, *C*_deg_ as well as CSI should be chosen, with consideration given to their strengths and weaknesses (Fig. 6). We suggest that apart from the assessment of the overall contamination as well as determination of the potential ecological risk (RI), specificity of soils with O horizon requires the use of BGI (Kaste et al. 2011; Mazurek et al. 2017; Medyńska-Juraszek and Kabała 2012; Pająk et al. 2017). It should be mentioned that despite the obvious differences between BGI and other individual pollution indices, PCA diagrams created based on individual pollution index raw data show strong positive correlations between their values (Fig. 7). The main variability model component is connected with soil contamination as well as the sorption ability of O horizons (Błońska et al. 2016; Medyńska-Juraszek and Kabała 2012; Pająk et al. 2017; Zawadzki et al. 2007). Such situation may be a result of the similar method of calculation, which includes the presence of absolute values of contamination. Moreover, for forest soils, knowledge of heavy metal origin may be useful (Kawahigashi et al. 2011; Pająk et al. 2017); hence, we also suggest the use of EF.

**Fig. 8:**
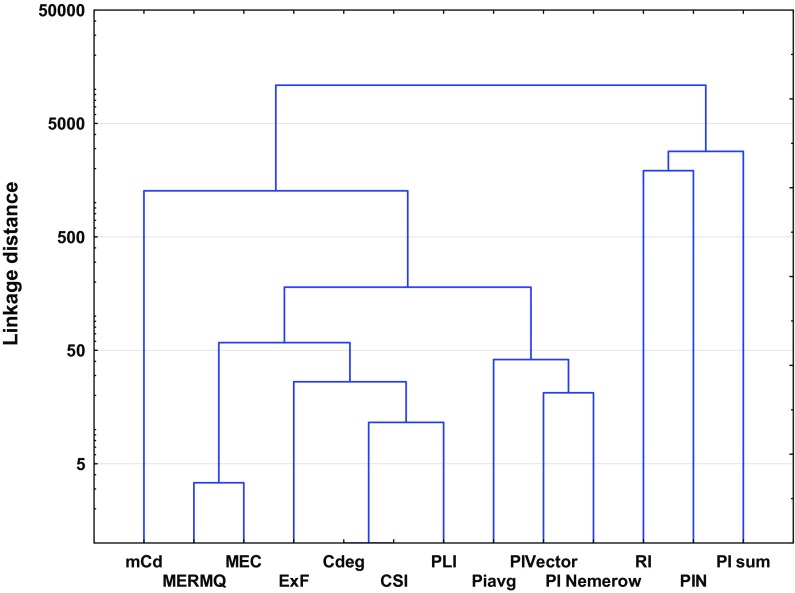
Ward’s hierarchical cluster analysis of the studied complex pollution indices based on urban soils

It is obvious that soils within urban areas are likely to be exposed to anthropogenic heavy metal pollution (Błońska et al. 2014; Markiewicz-Patkowska et al. 2005; Wei and Yang 2010). Enrichment by heavy metals may be a result of different industrial and commercial activity as well as historical pollution (Kowalska et al. 2016). Some studies have shown that soils in urban areas may be affected by enrichment with individual metals, which is often associated with the type of industry in the city and its surroundings (Ekwere et al. 2014; Elias and Gbadegesin 2011; Mazurek et al. 2017). It is also important in urban areas to compare the pre-industrial state of soil with present conditions (Błońska et al. 2014; Golyeva et al. 2014; Halecki and Gąsiorek 2015; Kowalska et al. 2016). In our opinion, a comprehensive approach to urban soil quality evaluation requires application of some individual indices to assess contamination with individual heavy metals (*I*_geo_, PI or *C*_f_). These indices show statistical similarities (Fig. 1), so they might to some extent be interchangeable, including in terms of their purpose, as well as their advantages and disadvantages. Further, EF should be applied in order to identify the origin of pollution (Gao and Chen 2012; Kowalska et al. 2016). Similar to farmland and forest soils, clusters created for complex pollution show two main groups; thus, the choice of an appropriate pollution index should include one of the ‘*PI*-indices’ or one of the following indices: mCd, ExF, MERMQ, MEC, *C*_deg_ or CSI. It should be noted that the latter cluster includes RI (Fig. 8). Nevertheless, because of the fact that RI values are able to recognize potential ecological risks (Table 1), we propose to use them regardless of any correlations with other indices within urban soil quality assessment processes.

### Geochemical background

Application of GB focuses on the reliability of the characterization and quantification of heavy metals in soil (Gąsiorek et al. 2017; Loska et al. 2004; Matschullat et al. 2000; Xu et al. 2015). Furthermore, choice of an appropriate GB plays an important role in the interpretation of heavy metal enrichment (Gałuszka and Migaszewski 2011; Varol 2011). A common problem regarding soils, sometimes due to the variable origin of parent material or parent materials (Waroszewski et al. 2017), is the question of which layer or horizon should be considered the GB (Kowalska et al. 2016). Various GBs (local and reference) could be applied in order to produce a more accurate examination of pollution index values. A crucial point is to recognize which GB should be used, and this may be dependent on the possibility of the contamination of individual soils/sites (Dung et al. 2013; Gałuszka 2007; Karim et al. 2015; Tomaškin et al. 2013; Varol 2011). Many biogeochemical questions are related to the application of appropriate RGB and LGB.

In general, RGB includes the concentration of heavy metals in UCC, LCC or mean heavy metal content in the topsoil (surface) horizons worldwide (Table 3) and has a relationship with the general geological reference level (Gałuszka 2007; Xu et al. 2015). Some papers focusing on the assessment of heavy metal concentration have applied RGB to compare current pollution with ‘pre-civilization’ ranges (e.g., Abrahim and Parker 2008; Kowalska et al. 2016). It should be mentioned that RGBs do not involve natural variability or natural heavy metal anomalies (Kabata-Pendias 2011; Tomaškin et al. 2013; Xu et al. 2015). Moreover, by using only RGB it is not always possible to recognize natural influences and anthropogenic heavy metal contamination (Gałuszka and Migaszewski 2011; Kowalska et al. 2016). Nevertheless, the use of RGB makes a great deal of sense for global models of heavy metal assessment or concerning regional and more difficult aspects of pollution (Karim et al. 2015; Matschullat et al. 2000). RGB allows information concerning soil quality evaluation to be considered at a global scale and allows comparisons beyond the local scale. Pollution indices that need RGB in their calculation can have a more multi-purpose character. Together with the rapid increase in urbanization and industrialization, significant inputs of human-derived substances deposited in and/or on soil profiles entail difficulties or make it impossible to determine the degree of pollution using only RGB (Karim et al. 2015; Lis and Pasieczna 1997; Reimann and Garret 2005; Zhou et al. 2015).

Considering the above-mentioned conditions, LGB is increasingly used (Kowalska et al. 2016; Mazurek et al. 2017). LGB includes heavy metal accumulation in the most pristine sites or heavy metal composition in rocks and the mean content of sample populations (Chen et al. 2016; Evseev and Krasovskaya 2015; Reiman and Garret 2005; Tomaškin et al. 2013). LGB considers a certain degree of human impact (Karim et al. 2015). Furthermore, it allows comparison of pollutant concentration in the upper layers with subsoil horizons of the same soil profile and also takes into account the heavy metal cycle (Kabata-Pendias 2011; Kowalska et al. 2016). LGB is also recommended for individual sites under the influence of natural processes (Kierczak et al. 2016; Matschullat et al. 2000). LGB use is suggested especially when anthropogenic impact and high levels of contamination are suspected. However, LGB may vary significantly across lithogenic conditions and its level should be assessed within pedologically and geologically homogenous areas.

It has been established that RGB as well as LGB values should be used to obtain complete information (Gąsiorek et al. 2017; Kowalska et al. 2016; Mazurek et al. 2017; Reiman and Garret 2005). A holistic approach is meaningful in order to avoid confusion during the choice of soil quality evaluation algorithm. Independent of the background used and degree of heavy metal pollution, indices might not always be able to show environmental threats in an accurate way, as the threshold level of toxicity to human health is still not clear and is highly individual (Dung et al. 2013; Gałuszka 2007; Matschullat et al. 2000). Hence, we suggest considering a comprehensive method that includes the application of indirect and direct data (RGB and LGB, respectively) as well as absolute heavy metal content.

## Conclusions

Calculation of indices characterized by various features helps to find or establish the right theoretical basis for appropriate interpretation of soil conditions. In this paper, 18 different indices of pollution have been reviewed and initially divided into individual and complex groups. Pollution indices include the newly introduced Biogeochemical Index (BGI), which is significant for O horizon quality assessment. Nevertheless, pollution indices are seemingly characterized by several similarities. Hence, they may be divided into five additional groups based on their different purposes, and into three groups based on the method of calculation. Statistical analysis confirmed some differences and similarities between the studied indices. Furthermore, a comparison of the strengths and weaknesses of each index has been made. This approach allows us to point out the specific limitations of each index in various configurations. According to the authors, among the individual pollution indices *I*_geo_ and EF are considered the most useful and universal, whereas of the complex pollution indices RI and CSI have been found to be the most important. To appropriately understand the degree of pollution, the choice of proper index is key, and both the soil use and the purpose of pollution indices calculation should be considered. Specific selection of pollution indices, based on their purposes, advantages and disadvantages, can be applied to comprehensive assessment of soil conditions under various uses—farmland, forest and urban areas.

Some of the pollution indices require the determination of the geochemical background (GB). Establishing an appropriate GB plays an important role and should be based on soil- and site-specific criteria as well as the purpose and scale of the heavy metal assessment process. We suggest a comprehensive approach based on the application of local and reference GBs to assess the quality of a given soil sample. A holistic approach is advisable in order to avoid confusion and uncertainty during soil quality evaluation.

## Electronic supplementary material

Below is the link to the electronic supplementary material.

Supplementary material 1 (DOCX 18 kb)  We forgot to add the financial support source. We will be grateful if you would add the following sentence: Acknowledgements: This Research was financed by the Ministry of Science and Higher Education, Republic of Poland.  
