# Supplementary material for: Pollution indices as useful tools for the comprehensive evaluation of the degree of soil contamination–A review
Source: Environ Geochem Health. 2018 Apr 5;40(6):2395–420. doi: 10.1007/s10653-018-0106-z (PMC6280880; doi:10.1007/s10653-018-0106-z)
Supplement: Supplementary file 1 — Supplementary material 1 (DOCX 18 kb) We forgot to add the financial support source. We will be grateful if you would add the following sentence: Acknowledgements: This Research was financed by the Ministry of Science and Higher Education, Republic of Poland. [file 10653_2018_106_MOESM1_ESM.docx]

Supplementary material

Table S1. Soil quality according to Geoaccumulation Index *I_geo_* values

| Class | Values of *I_geo_* | Soil quality |
| --- | --- | --- |
| 0 | *I* ≤ 0 | unpolluted |
| 1 | 0-1 | unpolluted to moderately polluted |
| 2 | 1*-*2 | moderately polluted |
| 3 | 2-3 | moderately to highly polluted |
| 4 | 3-4 | highly polluted |
| 5 | 4-5 | highly to extremely high polluted |
| 6 | 5-6 | extremely high polluted |

Table S2. Contamination classes of Single Pollution Index (*PI*)

| Class | Value of *PI* | Soil pollution |
| --- | --- | --- |
| 1 | PI <1 | absent |
| 2 | 1< PI< 2 | low |
| 3 | 2< PI< 3 | moderate |
| 4 | 3< PI< 5 | strong |
| 5 | PI >5 | very strong |

Table S3. Table S3. Categories of Enrichment Factor (*EF*)

| *EF* Value | Enrichment of soil |
| --- | --- |
| < 2 | deficiency to minimal enrichment |
| 2-5 | moderate enrichment |
| 5-20 | significant enrichment |
| 20-40 | very high enrichment |
| > 40 | extremely high enrichment |

Table S4. Pre-industrial reference level and toxicity response (µg∙g^-1^)

| Elements | Hg | Cd | As | Cu | Pb | Cr | Zn | Ni |
| --- | --- | --- | --- | --- | --- | --- | --- | --- |
|  | µg∙g^-1^ | | | | | | | |
| Pre-industrial reference level | 0.25 | 1.0 | 15 | 50 | 70 | 90 | 175 | 5 |
| Toxicity response | 40 | 30 | 10 | 5 | 5 | 2 | 2 | 5 |

Table S5. Contamination Factor (*C_f_* ) and Degree of Contamination (*C_deg_*) interpretation

| *C_f_* value | Contamination | *C_deg_* value | Contamination |
| --- | --- | --- | --- |
| < 1 | low contamination | < 8 | low degree of contamination |
| 1-3 | moderate contamination | 8-16 | moderate degree of contamination |
| 3-6 | considerable contamination | 16-32 | considerable degree of contamination |
| > 6 | very high contamination | > 32 | very high degree of contamination |

Table S6. Biogeoaccumuation Index (*BGI*) degrees

| Value of *BGI* | Degree of *BGI* |
| --- | --- |
| < 1 | low |
| 1-5 | apparent |
| 5-10 | moderate |
| 10-15 | considerable |
| 15-20 | high |
| > 20 | very high |

Table S7. Nemerow Pollution Index (*PI_Nemerow_)* soil pollution classes

| Class | I | II | III | IV | V |
| --- | --- | --- | --- | --- | --- |
|  | ≤ 0.7 | 0.7-1 | 1-2 | 2-3 | ≥ 3 |
| Quality of soil | Clean | Warning limit | Slight pollution | Moderate pollution | Heavy pollution |

Table S8. Contamination categories of Pollution Load Index (*PLI*)

| Value of *PLI* | Pollution status |
| --- | --- |
| < 1 | denote perfection |
| 1 | only baseline levels of pollution |
| > 1 | deterioration of soil quality |

Table S9. Interpretation of Background Enrichment Factor (*PIN)*

| Classes | *PIN* values | Contaminants |
| --- | --- | --- |
| 1 | 0-7 | clean |
| 2 | 7-95.1 | trace |
| 3 | 95.1-518.1 | lightly |
| 4 | 518.1-2548.5 | contaminant |
| 5 | ≥ 2548.8 | highly |

Table S10. ERM and ERL values given by Long et al. (1995)

| Element | ERM | ERL |
| --- | --- | --- |
| As | 70 | 8.2 |
| Cd | 9.6 | 1.2 |
| Cr | 370 | 81 |
| Cu | 270 | 34 |
| Pb | 218 | 46.7 |
| Hg | 0.71 | 0.15 |
| Ni | 51.6 | 20.9 |
| Ag | 3.7 | 1.0 |
| Zn | 410 | 150 |

ERM-effects range median; ERL-effects range low

Table S11. Weight of each heavy metal according to Pejman et al. (2015)

| Element | Computed weight |
| --- | --- |
| Cu | 0.075 |
| Zn | 0.075 |
| Cr | 0.134 |
| Ni | 0.215 |
| Pb | 0.251 |
| Cd | 0.25 |

Table S12. Classification of Contamination Security Index (*CSI*)

| Classification of *CSI* | Contamination severity |
| --- | --- |
| < 0.5 | uncontaminated |
| 0.5-1 | very low severity |
| 1-1.5 | low severity |
| 1.5-2 | low to moderate severity |
| 2-2.5 | moderate severity |
| 2.5-3 | moderate to high severity |
| 3-4 | high severity |
| 4-5 | very high severity |
| > 5 | ultra-high severity |

Table S13. The Probability of Toxicity Index interpretation (*MERMQ*)

| *MERMQ value* | Risk level | Probability of toxicity (%) |
| --- | --- | --- |
| < 0.1 | low | 9 |
| 0.1-0.5 | medium | 21 |
| 0.5-1.5 | high | 49 |
| > 1.5 | very high | 76 |

Table S14. Grades of Potential Ecological Risk (*RI*)

| *RI* | Potential Ecological Risk |
| --- | --- |
| < 90 | low |
| 90-180 | moderate |
| 180-360 | strong |
| 360-720 | very strong |
| ≥ 720 | highly-strong |

Table S15. Classification of Modified Degrees of Contamination (*mCd*)

| Values of *mCd* | Degree of contamination |
| --- | --- |
| < 1.5 | very low |
| 1.5-2 | low |
| 2-4 | moderate |
| 4-8 | high |
| 8-16 | very high |
| 16-32 | extremely high |
| > 32 | ultra-high |
